# Impact of Nitric Oxide-Release Kinetics on Antifungal Activity

**DOI:** 10.3390/jof10050308

**Published:** 2024-04-24

**Authors:** Quincy E. Grayton, Ivie L. Conlon, Christopher A. Broberg, Mark H. Schoenfisch

**Affiliations:** 1 Department of Chemistry, University of North Carolina at Chapel Hill, Chapel Hill, NC 27599, USA; quincyd@live.unc.edu (Q.E.G.); cbroberg@unc.edu (C.A.B.); 2 Eshelman School of Pharmacy, University of North Carolina at Chapel Hill, Chapel Hill, NC 27599, USA

**Keywords:** *Candida albicans*, *Candida auris*, *Cryptococcus neoformans*, *Aspergillus fumigatus*, nitric oxide, antifungal

## Abstract

Pathogenic fungi are an increasing health threat due to the rise in drug resistance. The limited number of antifungals currently available and growing incidence of multi-drug-resistant fungi has caused rising healthcare costs and a decreased quality of life for patients with fungal infections. Nitric oxide (NO) has previously been shown to act as an antimicrobial agent, albeit with a limited understanding of the effects of the NO-release kinetics against pathogenic fungi. Herein, the antifungal effects of four nitric oxide-releasing small molecules were studied against the pathogenic fungi *Candida albicans*, *Candida auris*, *Cryptococcus neoformans*, and *Aspergillus fumigatus*, to demonstrate the broad-spectrum antifungal activity of NO. A bolus dose of NO was found to eradicate fungi after 24 h, where nitric oxide donors with shorter half-lives achieved antifungal activity at lower concentrations and thus had wider selectivity indexes. Each NO donor was found to cause a severe surface destruction of fungi, and all NO donors exhibited compatibility with currently prescribed antifungals against several different fungi species.

## 1. Introduction

Pathogenic fungi are an increasing global health threat with fungal infections such as candidiasis, meningitis, and aspergillosis affecting over one billion people annually [1,2,3]. Many pathogenic fungi are opportunistic pathogens, affecting critically ill patients and those with underlying conditions such as HIV/AIDS, cancer, transplants, chronic respiratory diseases, and liver or kidney diseases [2]. The extent of invasive fungal infections is difficult to quantify, widely underdiagnosed, and underreported, and research and development is poorly funded [2,4,5]. The World Health Organization (WHO) recently published the fungal priority pathogens list, representing the first global effort to prioritize fungal pathogens [6]. The list categorizes fungi based on their priority (e.g., critical, high, and medium) and health risk, such as potential to cause invasive and acute systemic fungal infections, along with challenges related to treatment and resistance. The four critical priority group pathogens were identified to be *Aspergillus fumigatus*, *Cryptococcus neoformans*, *Candida auris*, and *Candida albicans* [6].

*Aspergillus fumigatus* (*A. fumigatus*) is an environmental mold that causes the respiratory illness aspergillosis, which increasingly shows resistance to azoles, a class of antifungals that are used as a first-line-of-defense treatment [7,8]. Azole resistance is perpetuated, in large part, due to fungicides employed globally to treat plant infection. In fact, strains of azole-resistant *A. fumigatus* that cause aspergillosis in humans were found to have the same resistance gene markers as environmentally resistant strains [8]. *Cryptococcus neoformans* (*C. neoformans*) is a yeast that is also found within the environment, and clinical infections have steadily increased due to the increased use of immunosuppressive medications. The fungus affects the respiratory system and central nervous system, causing cryptococcosis, cryptococcal meningitis, and cryptococcaemia [9]. The severity of cryptococcosis is demonstrated in patients with HIV, resulting in a mortality rate between 41 and 60% following infection [9]. In addition to its high mortality rate, *C. neoformans* is innately resistant to echinocandins, a class of antifungals that inhibit cell wall synthesis, immediately limiting the options of care to antifungals such as amphotericin B and azoles, which can cause drug–drug interactions and/or hepatotoxicity [10,11]. More than 80% of reported fungal infections are caused by *Candida* yeast strains (i.e., *C. albicans*, *C. glabrata*, *C. auris*), of which over 30 species have been identified to cause invasive infections (candidiasis) of the blood, heart, central nervous system, eyes, bones, and internal organs [3]. Invasive candidiasis is a serious nosocomial infection that especially affects critically ill and immunocompromised patients [6]. Candidiasis presents a serious global health challenge, as it is often multi-drug-resistant, is frequently misidentified during laboratory testing, and easily spreads between patients [6,12].

Currently, the scope of treatment for fungal infections is limited, with only four classes of antifungals routinely employed, including polyenes, azoles, echinocandins, and pyrimidines [6]. Polyenes interact with sterols in cell membranes to form channels through which small molecules leak out from fungal cells to the extracellular space [13]. Azoles interfere with the production of ergosterol, which is required for the synthesis and structure of fungi cell membranes [13]. Similarly, echinocandins inhibit the synthesis of β-glucan, which is a crucial component of the fungal cell wall [14]. Finally, pyrimidines inhibit synthesis of fungal DNA and RNA [15]. Antifungal selection and use are limited, as antifungals are generally highly toxic and result in off-target effects due to the similarities between mammalian and fungal cells, possess poor pharmacokinetic and pharmacodynamic properties, and can have undesirable drug–drug interactions [6,16]. For example, the polyene amphotericin B interacts with ergosterol in the fungal cell membrane, leading to loss of osmotic balance and cell death. However, it can also bind to cholesterol in human kidney and liver cells, resulting in severe nephrotoxicity [13,17,18]. Additionally, the structural complexity of fungi (i.e., spores, hyphae, mycelia) often impairs pharmaceutical interventions, impacting the degree and longevity of the infection [19,20].

Chronic use of antifungals compounded with prophylactic use has driven the incidence of multi-drug-resistant fungi [5]. Resistance to the few drugs currently available can eliminate treatment options, diminishing patient care outcomes and increasing therapeutic failure [10]. In 2018, over 90% of blood laboratory samples of *C. auris* were resistant to at least one antifungal, whereas 30% were resistant to at least two antifungals [12,21,22]. The issue of drug resistance is intensified by the slow development of antifungal drugs, with only a few compounds approved in the past fifty years [23,24]. As a result of emerging antifungal resistance and high toxicity, the need for new therapeutics to treat fungal infections is critical.

Nitric oxide (NO) is an endogenous signaling molecule that is intricately involved in host pathogen defense [25,26]. Indeed, NO mediates multiple mechanisms of action by forming reactive byproducts (e.g., oxidative and nitrosative stressors) that damage DNA and nitrosate thiols and destroy microbial membranes [26,27]. A number of chemical NO donors including small molecules and macromolecular systems have been proposed as treatments for pathogenic fungi [27,28,29]. Stasko et al. reported on the benefits of a topical NO-releasing silica nanoparticle to eradicate a panel of fungi, including *Trichophyton rubrum*, *Trichophyton mentagrophytes*, *Epidermophyton floccosum*, *Fussarium*, *Malassezia furfur*, and *C. albicans* [27]. Indeed, the NO-release system elicited a >4 log reduction in colony-forming units per mL for each fungal species within 24 h, implicating NO as a rapid and effective antifungal treatment [27]. In another study by Madariaga-Venegas et al., NO-releasing aspirin demonstrated antifungal and antibiofilm effects against *C. albicans*, and Vargas-Cruz et al. employed a nitroglycerin–citrate–ethanol catheter lock solution to successfully eradicate *C. auris* biofilms in catheter lumens [30,31].

Our lab previously utilized small-molecule NO donors in exploratory studies toward new antimicrobial therapies [32,33,34]. Small-molecule NO donors present a unique opportunity to deliver large doses of NO (5–7 µmol mg^−1^) to quickly eradicate pathogens. Previously, the small-molecule diethylenetriamine/NO (DETA/NO) demonstrated antifungal activity against six strains of *Candida* species and had synergistic activity when treated in combination with azole antifungals [35]. However, the antifungal effects of small-molecule NO donors with varying release kinetics have yet to be evaluated. Comparing trends in efficacy versus payload and half-life of NO release against multiple fungal species will inform the development of novel, broad-spectrum NO donors as potential antifungal agents. Therefore, in this work, we evaluated small-molecule NO donors with varying release kinetics to elucidate the effects of NO on the four pathogenic fungi indicated as a critical threat by the WHO (i.e., *C. albicans*, *C. auris*, *C. neoformans*, and *A. fumigatus*) and evaluated their potential utility for combination therapy with existing antifungals.

## 2. Materials and Methods

### 2.1. Materials

Diethylenetriamine (DETA), spermine (SPER), and bis(3-aminopropyl) amine (DPTA) were purchased from Sigma-Aldrich (St. Louis, MO, USA). Methyl tris diazeniumdiolate (MD3) was a gift from Vast Therapeutics (Morrisville, NC, USA). Gibco RPMI 1640 Medium and common laboratory salts and solvents were purchased from Thermo Fischer Scientific (Fair Lawn, NJ, USA). Unless otherwise specified, chemicals were used as received without further purification. Distilled water was purified to a resistivity of 18.2 MΩ-cm and a total organic content of ≤6 ppb using a Millipore Milli-Q UV Gradient A10 system (Bedford, MA, USA). Phosphate-buffered saline (PBS) was 10 mM at pH 7.4, unless otherwise specified. The medium 2× RPMI, buffered to pH 7.4 using a 0.165 M solution of 3-(*N*-morpholino)propanesulfonic acid (MOPS) and 2% solution of glucose, was used for susceptibility, dose-dependent, and checkerboard assays, SEM, and confocal microscopy unless otherwise stated. Yeast extract peptone dextrose (YPD) and Sabouraud dextrose agar (SDA) and broth (SDB) were obtained from Becton, Dickinson, and Company (Franklin Lakes, NJ, USA). EpiAirway kits (AIR-100) consisting of EpiAirway cultures, assay medium, trans-epithelial electrical resistance (TEER) buffer, MTT (3-4,5-dimethylthiazol-2yl)-2,5-diphenyltetrazolium bromide) diluent, MTT concentrate, and MTT extractant were purchased from MatTek (Ashland, MA, USA). Argon, nitrogen (N_2_), and nitric oxide (NO) gas cylinders were purchased from Airgas National Welders (Durham, NC, USA). Vero E6 cells were purchased from ATCC and cultured in Dulbecco’s Modified Eagle Medium (DMEM) supplemented with 10% fetal bovine serum (FBS) and 1 wt% penicillin/streptomycin/amphotericin B (P/S/A). Cells were grown in a 5% CO_2_ incubator at 37 °C.

### 2.2. Antifungals

The amphotericin B solution (250 µg mL^−1^ in deionized water) was obtained from Sigma-Aldrich (St. Louis, MO, USA), while miconazole nitrate powder was obtained from Sigma-Aldrich (St. Louis, MO, USA) and resuspended in sterile H_2_O. Caspofungin, fluconazole, 5-fluorocytosine, and butenafine powders were obtained from Cayman Chemical (Ann Arbor, MI, USA) and resuspended in DMSO, ethanol, sterile H_2_O, and DMSO, respectively. Antifungal drugs were diluted to a working concentration in the 2× RPMI 1640 medium.

### 2.3. Fungal Strains

The yeast strains *Candida albicans* (ATCC MYA-2876, ATCC 18804, ATCC 14053), *Candida auris* (MYA-5000, MYA-5001, MYA-5003), and *Cryptococcus neoformans* (ATCC208821, ATCC4566, ATCC4567), and *Aspergillus fumigatus* (ATCC 1022) spores, were purchased from ATCC. Reference yeast strains were kept in a cryoprotective medium in 20% glycerol at −80 °C. Reference fungal spores were stored as a lyophilized powder at room temperature. Yeast cultures were streaked on YPD agar and incubated at 37 °C overnight before being sealed with parafilm and stored at 4 °C. Yeast cultures were streaked every seven days to ensure viable cells. The *A. fumigatus* spores were placed on SDA, streaked with a PBS-soaked Puritan standard cotton-tipped sterile applicator obtained from VWR (Radnor, PA, USA), and left at room temperature to sporulate for three weeks. Once spores were present, verified via light microscopy, 3 mL of PBS was added to the plate and a PBS-soaked Puritan standard cotton-tipped sterile applicator was used to gently remove spores and a pipette was used to transfer the solution to a sterile tube. A hemocytometer was used to count spores and diluted to a concentration of 2 × 10^6^ spores mL^−1^ and placed at 4 °C until use.

### 2.4. Synthesis of Small-Molecule NO-Releasing Compounds

To synthesize NO-releasing DPTA, DETA, and SPER, the organic bases (DPTA, DETA, or SPER) were dissolved in anhydrous acetonitrile (10 mL) at 33.3 mg mL^−1^. The solution was purged with argon six times (three 10 s purges, followed by three 10 min purges) at 100 psi inside a stainless steel Parr bomb with Teflon lining. The solution was pressurized to 290 psi with NO. After 3 days, the solution was purged with argon six times (three 10 s purges, followed by three 10 min purges) at 100 psi to remove unreacted NO. The resulting NO-releasing compounds were precipitated in cold diethyl ether, collected via centrifugation, dried under a vacuum, and stored in parafilmed vials at −20 °C.

### 2.5. Characterization of NO Release

Nitric oxide release was evaluated using a Sievers 280i chemiluminescence nitric oxide analyzer (NOA) (Boulder, CO, USA). The NOA was calibrated with air passed through a zero-NO filter (0 ppm NO) and 25.87 ppm of NO calibration gas (balance N_2_) prior to the analysis. The NO-releasing small molecules (1 mg) were dissolved in 30 mL of deoxygenated PBS at 37 °C. The solution was purged with nitrogen gas at a rate of 200 mL min^−1^, acting as a carrier gas, carrying liberated NO to the instrument. The analysis was stopped when NO levels fell below the quantitation limit of the instrument (10 ppb NO).

### 2.6. Susceptibility Assays

The minimum inhibitory concentration (MIC) of the NO-releasing compounds, scaffolds, and antifungals was determined using the broth microdilution method adapted from the CLSI M27 Reference Method [36]. Nitric oxide-releasing compounds and scaffolds were dissolved in 2× RPMI and immediately pH corrected to 7.4 using 1 M HCl, while antifungals were dissolved in the 2× RPMI medium and used directly. A 96-well plate format was used and included positive (yeast-containing medium without treatment) and negative (blank medium) controls. Before each experiment, yeast colonies from an inoculated YPD plate were suspended in PBS (5 mL) in a sterile tube. Tubes were vortexed and the solution was diluted to a concentration equivalent to McFarland 0.5. The MIC was established as the lowest concentration with no visible growth after a specific amount of time. For yeast strains *C. albicans* and *C. auris*, MIC values were determined after 24 h, while *C. neoformans* MIC values were determined after 72 h. Nitric oxide-releasing compounds and antifungals were tested over a range of concentrations, from 0.0039 mg mL^−1^ to 10 mg mL^−1^ and 0.0002 mg mL^−1^ to 4 µg mL^−1^, respectively. Breakpoints for antifungals were determined according to the European Committee on Antimicrobial Susceptibility Testing [37].

To establish MIC values for sporulating fungi, EUCAST Definitive Document E.Def.9.4 was used as a guideline [37]. The MIC values were assessed based on the lowest concentration of the compound that showed no visible growth of spores or hyphae. Sterile H_2_O (50 µL) was added into each well in columns 2–12 of a 96-well plate. NO-releasing compounds were dissolved in sterile H_2_O, immediately pH corrected to 7.4, and pipetted (100 µL) into the first column for a total of three technical triplicates. They were 2-fold serially diluted across the 96-well plate to obtain a final concentration ranging from 5 mg mL^−1^ to 0.002 mg mL^−1^. A fungi solution of 2 × 10^5^ spores mL^−1^ in sterile H_2_O (50 µL) was directly added on top of each well. After 24 h, 2× RPMI was added to the plate, and the plate was read after 48 h to establish the MIC.

### 2.7. Checkerboard Assays

Checkerboard assays were used to establish synergy between NO-releasing compounds and antifungals following a previously published protocol [38]. Briefly, stock solutions of the NO-releasing drugs and antifungals were prepared in the 2× RPMI 1640 medium. The 2× RPMI 1640 medium (100 µL) was added to all wells of a 96-well plate. The stock solutions containing the NO-releasing drugs were neutralized to pH 7.4, dispensed in row A (100 µL), and 2-fold serially diluted down the plate to row G. Then, 100 µL of the antifungal drug solutions was dispensed into each well of column 12. A 1:2 serial dilution was performed across the plate from column 12 to 2, resulting in concentrations of antifungals ranging from 0.5 to 250 µg mL^−1^. Lastly, 100 µL of 10^6^ CFU mL^−1^ yeast was added to each well in the plate. The plate was incubated at 37 °C and read after 24 h for *C. albicans* (MYA-2876) and *C. auris* (MYA-5001) and 48 h for *C. neoformans* (ATCC208821). The fractional inhibitory concentration (FIC) index for each drug combination was calculated using the following equation: ∑FIC = FIC_A_ + FIC_B_, where FIC_A_ is the concentration of the NO-releasing compound in a well divided by the MIC of that compound, and FIC_B_ is the concentration of the antifungal drug in the same well divided by the MIC of that drug. An FIC value less than or equal to 0.5 was regarded as synergistic, 0.5–1 as additive, 1–4 as indifferent, and greater than 4 as antagonistic [39].

To assess if the NO-releasing compounds affected the susceptibility of *A. fumigatus* spores to antifungal drugs, the yeast checkerboard assay was adapted. Spores were treated with NO-releasing compounds for 24 h in sterile H_2_O before treatment with antifungals. Briefly, 50 µL of sterile H_2_O was added to all wells in rows B through H of a 96-well plate. Aqueous solutions of the NO-releasing compounds were neutralized to pH 7.4, added (100 µL) to wells of row A, and two-fold serially diluted down the plate to row G. Spores were prepared the same as in the susceptibility assays described above, and 50 µL of spores at a concentration of 2 × 10^5^ spores mL^−1^ was added to rows A through G. The plate was incubated at room temperature. After 24 h, 100 µL of the 2× RPMI 1640 medium was added to columns 2–11 of a new 96-well plate. Antifungal solutions (100 µL) were added to each well in column 12 and 2-fold serially diluted across the plate through column 2. The solution from the antifungal plate (100 µL) was added to the corresponding wells of the treatment plate, and an additional 100 µL of spores was added to row H for antifungal treatment. The plate was read after 48 h.

### 2.8. Passaging Assays

To assess the ability of *C. albicans*, *C. auris*, and *C. neoformans* to develop resistance to the NO-releasing compounds MD3, SPER/NO, DPTA/NO, and DETA/NO compared to a commonly used antifungal (i.e., 5-fluorocytosine), passaging assays were performed. Briefly, a *C. albicans* (MYA-2876), *C. auris* (MYA-5001), or *C. neoformans* (ATCC208821) colony from an inoculated plate was suspended in 5 mL of SDB in separate sterile culture tubes and 1/10th of the MIC of each drug for the pathogens was directly added. The untreated and treated tubes were placed in a 37 °C incubator with shaking overnight. The next day, 5 mL of SDB was added to new separate sterile culture tubes. Then, 1/10th of the MIC of the drug was added in one tube and 100 µL of the overnight culture added to corresponding tubes, vortexed, and placed in a 37 °C incubator with shaking overnight. This process was repeated for 21 d. Cultures were streaked on a plate and tested for susceptibility to each compound every 7 d for the NO donors, and on days 1–7, 14, and 21 for 5-fluorocytosine.

### 2.9. Scanning Electron Microscopy

Colonies from an inoculated plate of *C. albicans* (MYA-2876), *C. auris* (MYA-5001), or *C. neoformans* (ATCC208821) were suspended in PBS in a sterile microcentrifuge tube and diluted to a concentration corresponding to an optical density (OD) of 1.0. The solution was diluted 1:1 in a 2.5 mL 2× RPMI 1640 medium for a total volume of 5 mL. The microbes were treated at 10× MIC of the NO-releasing compounds, spermine, or miconazole overnight in a 37 °C incubator. After 24 h, the pathogens were pelleted at 21,300 rcf for 2 min on an Eppendorf centrifuge (Enfield, CT, USA). The remaining solution was discarded, and the pellet was washed with 5 mL of PBS three times. The sample was incubated in 2.5% glutaraldehyde in PBS for 3 h at 4 °C, pelleted, and washed with 5 mL of PBS two times. A series of ethanol washes were used to dehydrate the sample. The sample was pelleted after a 10 min dehydration at room temperature in increasing ethanol concentrations of 30%, 50%, 70%, 90%, and 100%. After the final dehydration step, the sample was pelleted and allowed to air dry in the microcentrifuge tube. It was then sputter-coated with gold and palladium and viewed on a Hitachi S-4700 Cold Cathode Field Emission Scanning Electron Microscope (Chiyoda, Japan).

### 2.10. Confocal Microscopy

Colonies from an inoculated plate of *C. albicans* (MYA-2876) were suspended in PBS in a sterile microcentrifuge tube and diluted to a concentration corresponding to an OD of 0.5. The solution was diluted 1:1 in the 2× RPMI 1640 medium to achieve a total volume of 1 mL. The microbes were treated at 5× MIC of SPER/NO or MD3. Before imaging, 200 µL of calcofluor white, 200 µL of propidium iodide, and 10 µL of DAF2 diacetate were added to each well of a Nunc Lab-Tek II chamber slide and incubated for 60 min. Images of untreated and NO-treated *C. albicans* were taken after 24 h on an Andor Dragonfly Spinning Disk Confocal Microscope (Oxford Instruments; Carteret, NJ, USA). All confocal microscopy experiments were performed at 37 °C. Images were acquired with an HC PL APO 100×/1.40 OIL CS2 objective in three random spots at the top, middle, and bottom of each well, acquiring a 3 × 3 grid of images in each spot. The following lasers were used for excitation: 405 nm for calcofluor white, 488 nm for the fluorescent reaction product of DAF-2 and NO, and 561 nm for propidium iodide. These lasers were detected at 445 nm, 521 nm, and 594 nm, respectively, using a Zyla Plus 4.2MP sCMOS camera (Andor Technology; Belfast, UK).

### 2.11. Tissue Viability Assays

MatTek AIR-100 tissues were cultured and the MTT viability assay was conducted following MatTek protocols. The NO donors were dissolved in PBS (10 mM; pH 7.4) at 8 to 64 mg mL^−1^ and titrated with 0.1 to 5 M HCl to neutralize the pH to 7.4. Tissues were treated with 20 µL of the NO donor solution or PBS (control) applied directly to the apical tissue surface. After 24 h, tissues were rinsed three times with the TEER buffer and incubated with 300 µL of the MTT reagent (MTT diluent and concentrate) in a 24-well plate for 1.5 h at 37 °C. Tissue inserts were removed from the MTT solution, blotted dry on a paper towel, and incubated in 2 mL of the MTT extractant solution in a 24-well plate at room temperature overnight. At the end of the extraction period, the tissue inserts were discarded and 200 µL of the extractant solution was added to wells of a 96-well plate and the absorbance (Abs) of each well was measured at 570 nm, using 200 µL of a fresh extractant solution as a blank. A background reading at 650 nm was subtracted out for all samples. Relative tissue viability was determined using controls (PBS-treated cells) and blanks (extractant solution) as shown in Equation (1).

(1) $$\mathrm{Cell}\;\mathrm{Viability}=\frac{{\mathrm{Abs}}_{\mathrm{sample}}-{\mathrm{Abs}}_{\mathrm{blank}}}{{\mathrm{Abs}}_{\mathrm{control}}-{\mathrm{Abs}}_{\mathrm{blank}}}*100$$

Concentrations of material needed to inhibit tissue viability by 50% (IC_50_) were determined using a nonlinear regression (normalized response with variable slope) analysis in GraphPad Prism 10.1.1. The selectivity index (SI) was calculated for each NO donor by dividing the IC_50_ value by the MIC as shown in Equation (2).

(2) $$\mathrm{SI}=\frac{{\mathrm{IC}}_{50}}{\mathrm{MIC}}$$

### 2.12. Statistical Analysis

Nitric oxide-release measurements are presented as the average ± standard deviation from n = 3 NOA runs. Susceptibility, checkerboard, and cell viability assay results are depicted as the average ± standard deviation from n ≥ 3 biological replicates. SEM and confocal microscopy images shown are representative images from n ≥ 3 biological replicates.

## 3. Results

### 3.1. Nitric Oxide-Releasing Small Molecules Exhibit Broad Spectrum Antifungal Activity

**Figure 1:**
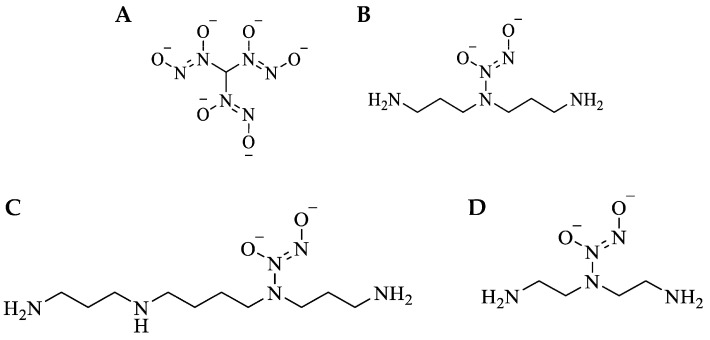
Small-molecule NO-releasing C- and N-diazeniumdiolates. (**A**) MD3: methyl tris diazenimdiolate, (**B**) DPTA/NO: bis-3-aminopropyl amine/NO, (**C**) SPER/NO: spermine/NO, (**D**) DETA/NO: diethylenetriamine/NO.

The four small-molecule NO donors investigated were previously identified by our lab as potent antimicrobial agents [40,41,42,43]. However, comparing the ability of these NO donors to eradicate pathogenic, planktonic fungi has yet to be evaluated. The NO donors investigated include methyl tris diazeniumdiolate (MD3, Figure 1A), bis(3-aminopropyl) amine/NO (DPTA/NO, Figure 1B), spermine/NO (SPER/NO, Figure 1C), and diethylenetriamine/NO (DETA/NO, Figure 1D). Release of NO from *N*-diazeniumdiolates and a mixture of NO, nitrous oxide (N_2_O), and/or nitroxyl (HNO) from *C*-diazeniumdiolates is the result of a proton-initiated reaction under physiological conditions [44,45]. The compounds tested herein were selected for their diverse NO-release kinetics and payloads (Table 1) to probe the most efficient NO-release profile (i.e., slow, medium, or fast NO release) for eradicating fungi. Each NO donor initially released a burst of NO, followed by prolonged release at lower concentrations for periods spanning 14–107 h (Figure S1).

The antifungal activities of the small molecules with and without NO were assessed by evaluating their minimum inhibitory concentration (MIC) against the four pathogens indicated as a critical priority by the WHO (i.e., *C. albicans*, *C. auris*, *C. neoformans*, and *A. fumigatus*) for being a major global health threat [36]. Additionally, antifungal activity of six FDA-approved antifungals, including fluconazole and miconazole (azole class), flucytosine (DNA/RNA inhibitor), butenafine (allylamine class), caspofungin (echinocandin class), and amphotericin B (polyene class), was tested against each fungi for comparison and to identify drug-resistant strains. Antifungal activity against *C. albicans*, *C. auris*, and *C. neoformans* was evaluated using the Clinical Laboratory Standards Institute (CLSI) Reference Method M27 broth dilution assay, whereas the antifungal activity against *A. fumigatus* spores was evaluated following guidelines in European Committee on Antimicrobial Susceptibility Testing (EUCAST) Definitive Document E.Def.9.4. Both CLSI and EUCAST state that any fungal isolate with an MIC greater than a specified concentration is considered resistant to that drug [37]. Table S1 summarizes the MICs of each commercial antifungal against the investigated fungi. The *C. albicans* (ATCC MYA-2876) and *C. auris* (ATCC MYA-5001) strains used in our study were resistant to fluconazole, with *C. auris* also being resistant to caspofungin. The *A. fumigatus* strain (ATCC 1022) was resistant to amphotericin B.

Three strains each of *C. albicans*, *C. auris*, and *C. neoformans* were chosen to account for possible genetic, metabolic, and phenotypic variability. Determining the susceptibility of different strains to potential therapeutics is helpful in determining pan-antifungal agents. The MIC values for the NO donors evaluated ranged from 3 µg mL^−1^ to 1250 µg mL^−1^, with corresponding total NO payloads spanning 0.45 to 262 µg mL^−1^ (Table 2). Of note, *A. fumigatus* spores were killed at comparable NO concentrations to the other non-sporulating fungi. The calculated NO dose from each donor required to elicit antifungal activity at the MICs indicates that the potency of the active NO moiety is clinically and therapeutically relevant to currently prescribed antifungals (i.e., in µg mL^−1^). Of note, the MICs for each backbone without NO were ≥20 mg mL^−1^ for *C. albicans* and *C. auris*, and between 2.5 and >20 mg mL^−1^ for *C. neoformans* (Table S2).

### 3.2. Resistance to NO Avoided at Repeated Sub-Lethal Dose Exposure

**Figure 2:**
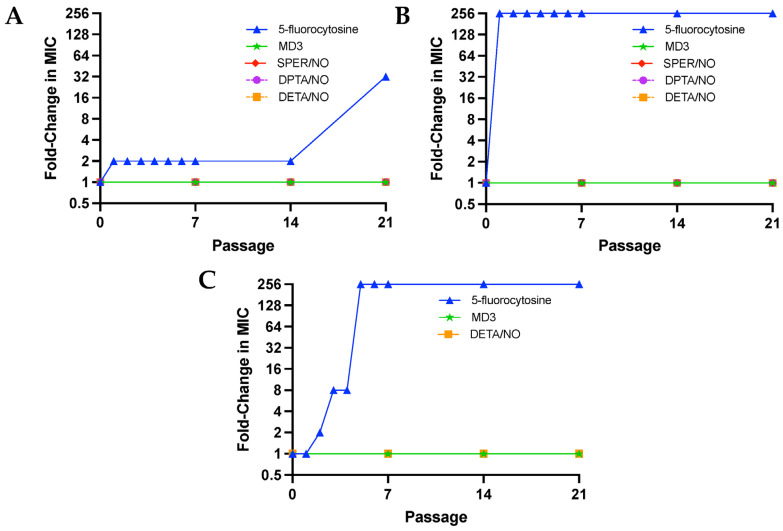
Fold-change in the minimum inhibitory concentration of NO donors and 5-fluorocytosine against (**A**) *Candida albicans* (ATCC MYA-2876), (**B**) *Candida auris* (ATCC MYA-5001), and (**C**) *Cryptococcus neoformans* (ATCC 208821) after serial passaging at 1/10th of the initial MIC.

Passaging assays were performed for 21 passages (each passage = 24 h), during which *C. albicans*, *C. auris*, and *C. neoformans* were exposed to sub-MIC levels (1/10th of MIC) of the NO donors or a traditional antifungal (i.e., 5-fluorocytosine) daily to evaluate the development of resistance. Given that all three strains of fungi were found to be susceptible to 5-fluorocytosine, it was selected as a control (Table S1), as the potential development of resistance to the antifungal could be observed for each of the pathogens. Of note, miconazole was also evaluated for use in such passaging assays; however, doses as low as 1/10th the MIC of miconazole consistently resulted in death of *C. albicans* after the second passage and was thus not utilized in subsequent studies. Susceptibility assays were performed after passage 7, 14, and 21 for the NO donors and after passage 1–7, 14, and 21 for 5-fluorocytosine. The earlier passages were included for 5-flurocytosine, as all fungal strains quickly acquired resistance to the antifungal, with MIC and MFC values increasing over 256-fold in *C. auris* and *C. neoformans* by passage 5 and 32-fold in *C. albicans* by passage 21 (Figure 2A–C). On the contrary, no change in MIC or MFCs was observed for MD3 and DETA/NO in all fungal strains regardless of passage. In addition, no changes in MIC or MFCs were observed for SPER/NO and DPTA/NO against *C. albicans* and *C. auris*. Passaging assays were not completed for *C. neoformans* with SPER/NO and DPTA/NO due to yeast death after the second passage from 1/10th to 1/40th of the MIC of both compounds, indicating that *C. neoformans* is particularly susceptible to *N*-diazeniumdiolate NO donors with short half-lives (<3 h) and large initial NO fluxes. Additionally, passing assays were not conducted for *A. fumigatus* spores, as spores are grown on agar instead of in broth, so a comparable assay could not be completed.

### 3.3. Nitric Oxide Treatment Is Compatible with Current Antifungals

Checkerboard assays were employed to evaluate the effects of NO donors on the activity of different classes of currently marketed antifungals (i.e., caspofungin, fluconazole, 5-fluorocytosine, amphotericin B, butenafine, and miconazole). The sum of fractional inhibitory concentrations (∑FICs) was used to determine the nature of in vitro drug interaction (i.e., if the combination treatment was synergistic, additive, indifferent, or antagonistic). Of importance, no interactions were found to be antagonistic, regardless of the NO compounds and antifungals tested, and most interactions were found to be indifferent (Table 3), demonstrating that the combinations between NO and antifungals do not negatively affect the antifungal mechanisms of action. Synergistic (∑FIC ≤ 0.5) and additive (0.5 < ∑FIC ≤ 1) interactions were observed with at least three different NO compounds for *C. albicans*, *C. auris*, and *C. neoformans*. The three microbes demonstrated synergy between SPER/NO and miconazole, suggesting that a large burst of NO, as is characteristic for NO donors with shorter half-lives, may be optimal to see synergistic activity. Overall, these data show that NO-releasing small molecules are synergistic or additive with miconazole and butenafine, two common topical antifungal drugs. No synergistic or additive interactions were observed against *A. fumigatus* with any of the antifungals evaluated.

### 3.4. Reduced Hyphae Formation and Surface Morphology Changes

**Figure 3:**
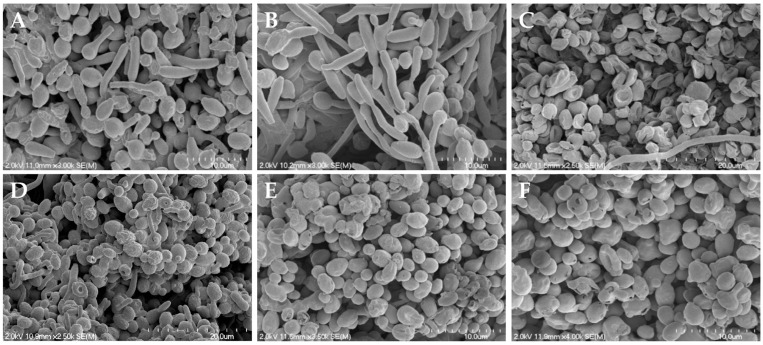
Scanning electron micrographs of (**A**) untreated *C. albicans* and *C. albicans* treated with (**B**) miconazole, (**C**) MD3, (**D**) SPER/NO, (**E**) DPTA/NO, and (**F**) DETA/NO at 10× the MIC for each compound. Images are representative of n ≥ 3 separate experiments.

**Figure 4:**
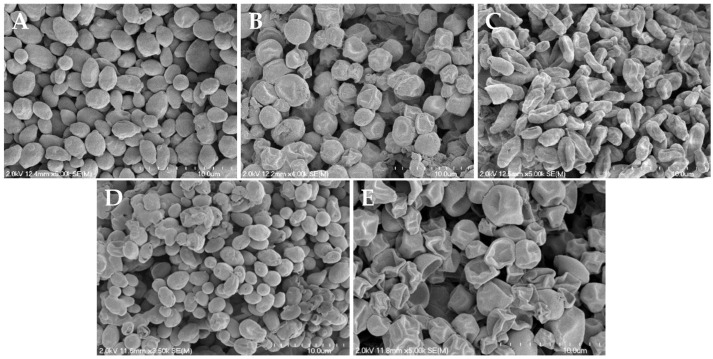
Scanning electron micrographs of (**A**) untreated *C. auris* and *C.auris* treated with (**B**) MD3, (**C**) SPER/NO, (**D**) DPTA/NO, and (**E**) DETA/NO at 10× the MIC for each compound. Images are representative of n ≥ 3 separate experiments.

As synergistic and additive effects were observed for *C. albicans*, *C. auris*, and *C. neoformans* when treated with the NO donors and butenafine or miconazole, two antifungals that impact fungal cell membranes, scanning electron microscopy (SEM) was utilized to visualize any physical impact of NO on the fungal envelopes. Microbes were exposed to each NO donor for 24 h in a 1:1 PBS:RPMI medium, and then dehydrated to enable viewing by SEM. As shown in Figure 3, Figure 4 and Figure S2, evaluation by SEM revealed that regardless of the NO donor employed, NO exposure significantly impacted the surface morphology of *C. albicans*, *C*. *auris*, and *C. neoformans*, respectively, as demonstrated by the increase in wrinkles and holes in the fungi after treatment with NO.

As shown in Figure 3A, untreated *C. albicans* readily form hyphae throughout the sample. While treatment with miconazole, a common antifungal that impairs the composition of the cell membrane, results in fungistatic action, it does not alter the presence of hyphae (Figure 3B). Upon treatment with the NO donors, however, hyphae formation was decreased or absent altogether (Figure 3C–F), with most yeast exhibiting a rough and disturbed oval topography. Susceptibility assays of NO-releasing compounds and controls (i.e., backbone) showed that the NO was solely responsible for *C. albicans* inhibition and eradication. In addition, when treated with the donor scaffold spermine without NO, hyphal formation was present with no cell death (Figure S3). Unlike with *C. albicans*, hyphae formation was not observed for untreated *C. auris* and *C. neoformans*, which displayed well-defined, oval cells (Figure 4A and Figure S2A). Upon treatment with NO donors, the morphology of *C. auris* and *C. neoformans* showed significant degradation, including a compromised topography and loss of the round structure (Figure 4B–E and Figure S2B–E).

**Figure 5:**
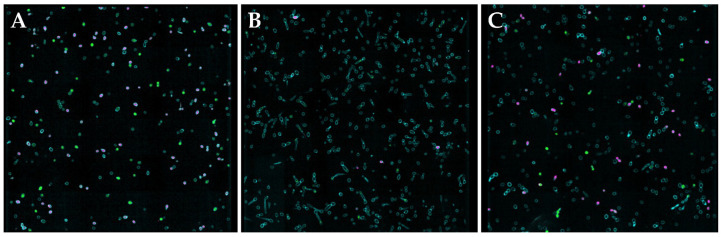
Confocal microscopy images of (**A**) untreated *C. albicans* and *C. albicans* treated with (**B**) MD3 and (**C**) SPER/NO at 5× their MIC for 24 h. Intracellular DAF-2 DA (green), calcofluor white (teal), and propidium iodide (pink) fluorescence. Images are representative of n ≥ 3 separate experiments.

In addition to decreased hyphae formation, the loss of fungal structural integrity was further investigated using confocal microscopy. Calcofluor white (CW) was used to stain the chitin of the cell wall in yeast buds and hyphae [18]. Dead cells and/or damaged membranes were observed using propidium iodide (PI) staining [32,46,47]. After treatment with MD3 (Figure 5B) and SPER/NO (Figure 5C) at 5× their MIC for 24 h, imaging of *C. albicans* showed a visual increase in PI fluorescence relative to the control sample (Figure 5A), indicating increased cell death after NO treatment. Additionally, a marked decrease in hyphae formation in treated samples relative to controls is observed by the CW staining, which stains the cell walls of both yeast buds and hyphae (Figure 5). Samples were also stained with 4,5-diaminofluorescein diacetate (DAF-2 DA) to evaluate intracellular NO concentrations [18,32,48]. In both the MD3 and SPER/NO images, the significant green fluorescence indicates increased NO levels within treated fungi (Figure 5B,C).

### 3.5. Tissue Viability and Determination of Susceptibility Indices

**Figure 6:**
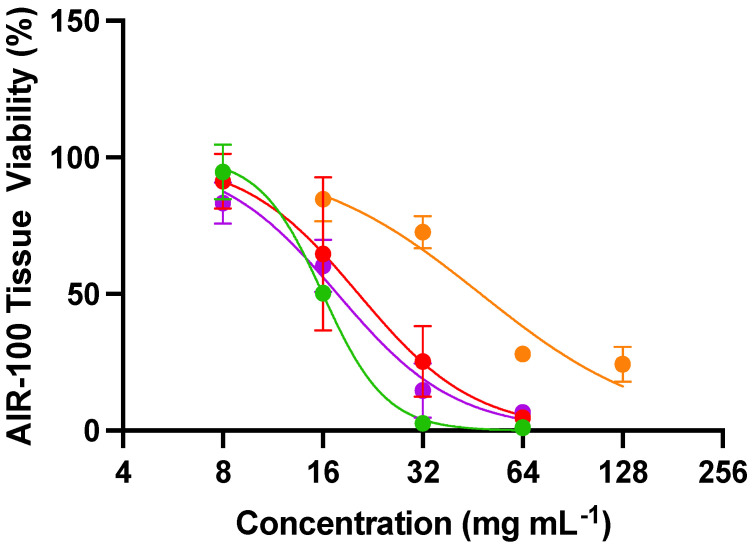
Viability curves for AIR-100 airway tissues from MatTek after 24 h apical treatment with 20 µL of MD3 (green), SPER/NO (red), DPTA/NO (purple), and DETA/NO (orange). Error bars represent the standard deviation from n = 3 biological replicates.

Toxicity of the NO donors was evaluated against an airway tissue model of the human respiratory epithelium to confirm that the efficacy observed in the above experiments is truly antifungal activity rather than a general toxic effect. The MatTek EpiAirway tissue (AIR-100) is a highly differentiated culture derived from primary human tracheal and bronchial epithelial cells grown at the air–liquid interface [49]. This tissue model was selected as *A. fumigatus* and *C. neoformans* can result in life-threatening invasive diseases of the respiratory system [50]. Tissue viability curves for AIR-100 tissues when treated for 24 h with the NO compounds are shown in Figure 6, and the concentrations of material needed to inhibit cell growth by 50% (IC_50_) are represented in Table S3. Cell toxicity was dependent on both the NO donor and its concentration, analogous to the antifungal activities. The slowest NO-releasing system (DETA/NO) exerted the least toxicity (IC_50_ of 48,000 µg mL^−1^), likely due to its long half-life of NO release (t_1/2_ = 22.5 h). The other NO donors exhibited similar toxicity profiles (IC_50_ between 16,000 and 20,000 µg mL^−1^). These IC_50_ values are significantly higher than the concentrations needed to exert antifungal activity. An antifungal should ideally have minimal toxicity to mammalian tissues, resulting in a wide selectivity index (SI) [51]. All of the NO donors had selectivity indices greater than 10, demonstrating their enhanced toxicity towards fungi compared to mammalian tissues (Table 4). Of the NO donors evaluated, DPTA/NO had the lowest selectivity indexes, with all values below 100 (Table 4). On the contrary, the other NO donors had much higher selectivity indexes, with several values above 1000, indicating a 3-log difference between the concentrations needed to exert antifungal activity and mammalian tissue toxicity. The NO donor MD3 had the highest average SI (491), thus representing the most promising antifungal evaluated herein.

## 4. Discussion

Nitric oxide is an endogenous, highly reactive signaling molecule involved in a number of key physiological processes, including vasodilation, wound healing, and host response to infection [27,52]. For example, immune cells such as macrophages and neutrophils upregulate the production of NO via inducible nitric oxide synthase (iNOS) in response to pathogens [53]. Although prior research has evaluated the use of gaseous NO to treat respiratory diseases, including hypoxic respiratory failure and persistent pulmonary hypertension in newborns, NO delivered in this manner can be both toxic at high concentrations and a presenter of safety concerns (e.g., methemoglobinemia) [25,54]. Thus, alternate strategies to deliver NO with less toxicity and adverse systemic effects are an active area of research. Both low-molecular-weight and macromolecular NO donors have been employed to study and target superficial fungal infections of the hair, skin, and nails, as well as treatment of burn infections caused by *C. albicans* [27,28,33,35,55]. We previously described the utility of an *N*-diazeniumdiolate-based NO-releasing xerogel to reduce *C. albicans* adhesion and biofilm formation [53].

In the present study, we sought to systematically evaluate the role of NO-release kinetics in the ability to eradicate pathogenic fungi more broadly. The data reported herein demonstrate the ability of NO, delivered by small-molecule NO donors, to effectively inhibit at least three species each of *C. albicans*, *C. auris*, and *C. neoformans*, and the sporulating fungi *A. fumigatus*. Each of the four NO donors tested had similar NO payloads, but diverse NO-release half-lives due to the stability of the *N*- or *C*-diazeniumdiolate structure, ranging from ~1 to ~20 h. Of the four NO donors, DETA/NO has the longest half-life, due to the terminal amine forming an ideally stabilized six-membered ring structure with the charged nitro groups [56]. Conversely, DPTA/NO and SPER/NO, having an additional carbon between amine groups in the linear structure, form slightly less stable seven-membered rings, with concomitantly faster NO release (i.e., shorter half-lives). Of note, DPTA/NO and MD3 both have ~3 h half-lives, yet remarkably different MICs, in part perhaps because MD3 is a *C*-diazeniumdiolate and may also release nitroxyl in addition to NO, which might explain the increased efficacy and antimicrobial activity of the donor [57].

Both MD3 and SPER/NO proved to be the most potent antifungal agents against *C. albicans*, *C. auris*, and *C. neoformans* (MIC ranges from 3 to 250 µg mL^−1^). Similar levels of MD3 and SPER/NO (compared to the levels needed for antifungal activity against *C. albicans*, *C. auris*, and *C. neoformans*) were needed for sporicidal activity against *A. fumigatus* (MIC of 70 µg mL^−1^). This broad-spectrum antifungal activity of NO is advantageous, as the persistence of fungal spores often leads to the failure of antifungal treatments [58,59]. Clearly, a rapid, burst NO release, characterized by a short half-life on the order of the lifespan of a fungus, results in the most effective killing and impacts active cellular division. Although the large initial burst of NO subsides by each NO donor’s half-life, the low doses of NO released over the subsequent several hours allows for continuous or sustained nitrosative and oxidative pressure on the fungi, effectively inhibiting propagation and resulting in cell death. Importantly, the concentration of NO required to eradicate each pathogen is significantly lower than the dose of the donor scaffold, as the active agent of these compounds is NO.

Others have previously reported that microbes such as *C. albicans* and *C. neoformans* possess flavohemoglobin, an NO dioxygenase that converts NO to nitrate as a detoxification strategy against NO produced endogenously [60,61,62]. The small-molecule NO donors have the potential to overcome such a microbial protection strategy by providing supraphysiologic doses of NO in a rapid fashion, effectively eradicating the pathogens. Of importance, unlike current antifungals, continuous sub-lethal treatment of NO did not induce resistance in any of the tested microbes, as evidenced by the passaging studies in this work, indicating that NO sourced via NO donors represents a promising strategy for combating fungal infections with reduced concern or risk for developing antimicrobial resistance. The effects of NO on the activity of current antifungals were evaluated using azole-resistant *C. albicans*, azole- and echinocandin-resistant *C. auris*, *C. neoformans*, and amphotericin B-resistant *A. fumigatus*. The NO compounds did not impact the activity of any of the antifungals evaluated and were found to be either synergistic or additive with miconazole, a widely used antifungal, suggesting that azole-resistant *C. albicans* could be successfully treated. Similar synergy was observed for NO donors and butenafine, an antifungal used to treat topical skin infections.

Scanning electron and confocal microscopies were used to visualize the effect of NO on each pathogen to provide mechanistic insight into potential modes of a synergistic effect. All NO donors tested interfered specifically with the morphological transformation between hyphae and buds in *C. albicans*. As the pathogenicity of *C. albicans* is directly related to the ability to change between the hyphae and bud morphologies, the NO donors appear to inhibit hyphae formation, a key virulence factor [19,63,64]. In contrast, miconazole, an azole drug that affects ergosterol synthesis and thus cell membrane composition, does not affect this morphological switch, further indicating that NO has the potential to be a potent antifungal against *C. albicans* relative to current drugs. In addition, the severe destruction of the yeast cell wall and membrane, cell death, and elevated levels of intracellular NO observed via SEM and confocal microscopy further demonstrates the potency of NO as an active antifungal agent. Tissue toxicity data indicated that all NO donors had selectivity indices above 10 for each fungal pathogen, demonstrating the ability of the NO donors to selectively exert antifungal activity without corresponding mammalian tissue toxicity. The *C*-diazeniumdiolate MD3 had the highest average selectivity index and thus represents a promising antifungal agent. Taken together, NO via small-molecule NO donors has the potential to eradicate fungal infections without the issues of resistance or tissue toxicity. Future studies should explore in vivo therapeutic efficacy and safety in established fungal infection models [27,65,66].

## 5. Conclusions

The continued rise in antifungal resistance suggests it is imperative to find a new class of antifungals. Four NO donors were used to assess antifungal activity as a function of NO payload and release kinetics. Our study revealed that NO is a potent, antifungal agent against *C. albicans*, *C. auris*, and *C. neoformans* and spores of *A. fumigatus*, with faster NO-releasing systems (i.e., SPER/NO) achieving more effective antifungal activity and having a wider selectivity index than the slower-releasing systems (i.e., DPTA/NO and DETA/NO). Additionally, the *C*-diazeniumdiolate MD3 proved to be more effective at eradicating fungi than the *N*-diazeniumdiolate NO donors and had wide selectivity indexes. As such, further development of NO donors for antifungal applications would benefit from a focus on fast NO-release kinetics and the use of *C*-diazeniumdiolates. As NO-releasing therapeutics have the potential to treat drug-resistant-strains of fungi without concern for promoting antimicrobial resistance and can overcome some of the toxicity concerns of current antifungals, they represent a promising alternative to conventional fungal treatments.

## Figures and Tables

**Table 1:** Nitric oxide-release properties of NO donors in phosphate-buffered saline (10 mM, pH 7.4, 37 °C) ^a^.

Material	[NO]_t_ (μmol mg^−1^) ^b^	[NO]_max_ (ppb mg^−1^) ^c^	t_1/2_ (h) ^d^	t_d_ (h) ^e^
SPER/NO	5.1 ± 0.2	7535 ± 222	1.1 ± 0.2	14.8 ± 2.9
MD3	5.0 ± 0.7	2800 ± 170	3.6 ± 0.8	42.8 ± 12.5
DPTA/NO	7.0 ± 1.1	2867 ± 211	3.7 ± 0.3	31.0 ± 3.3
DETA/NO	6.3 ± 0.6	735 ± 94	22.5 ± 3.3	107.6 ± 19.9

^a^ Error represents standard deviation for n ≥ 3 separate analyses. ^b^ Total NO released over full duration. ^c^ Maximum instantaneous NO concentration. ^d^ Half-life of NO release. ^e^ Duration of NO release.

**Table 2:** Minimum inhibitory concentrations (MICs) of NO donors against a panel of fungal isolates. MIC is reported as concentration of NO donor, with the corresponding calculated NO dose in parentheses ^a^.

Strains	MIC (µg mL^−1^) (MIC NO Dose (µg mL^−1^))			
	MD3	SPER/NO	DPTA/NO	DETA/NO
*Candida albicans*				
ATCC MYA-2876	60 (8.98)	125 (19.2)	500 (105)	125 (23.5)
ATCC 18804	40 (5.98)	150 (23.1)	625 (131)	625 (117)
ATCC 14053	150 (22.4)	250 (38.6)	625 (131)	625 (117)
*Candida auris*				
ATCC MYA-5000	30 (4.49)	125 (19.2)	625 (131)	625 (117)
ATCC MYA-5001	20 (2.25)	250 (38.6)	500 (105)	30 (5.65)
ATCC MYA-5003	40 (5.98)	125 (19.2)	310 (65.3)	310 (58.4)
*Cryptococcus neoformans*				
ATCC 208821	30 (4.50)	500 (76.8)	1000 (210)	250 (47.0)
ATCC MYA-4566	19 (2.99)	19 (3.08)	625 (131)	78 (14.7)
ATCC MYA-4567	20 (2.99)	78 (12.0)	625 (131)	310 (58.4)
*Aspergillus fumigatus*				
ATCC 1022	70 (10.5)	70 (10.7)	1250 (262)	320 (60.5)

^a^ Determined from n ≥ 3 experiments.

**Table 3:** Checkerboard assay results of NO donors and antifungals against *C. albicans* (ATCC MYA-2876), *C. auris* (ATCC MYA-5001), *C. neoformans* (ATCC 208821), and *A. fumigatus* (ATCC 1022). Synergistic (S), additive (A), and indifferent (I) classifications are shown in parentheses ^a^.

Antifungal	NO Donor	*C. albicans*	*C. auris*	*C. neoformans*	*A. fumigatus*
		∑FIC	∑FIC	∑FIC	∑FIC
Caspofungin	MD3	≥1 (I)	≥1 (I)	≥1 (I)	≥1 (I)
	SPER/NO	≥1 (I)	≥1 (I)	≥1 (I)	≥1 (I)
	DPTA/NO	≥1 (I)	≥1 (I)	≥1 (I)	≥1 (I)
	DETA/NO	≥1 (I)	≥1 (I)	≥1 (I)	≥1 (I)
Fluconazole	MD3	≥1 (I)	≥1 (I)	≥1 (I)	≥1 (I)
	SPER/NO	≥1 (I)	≥1 (I)	≥1 (I)	≥1 (I)
	DPTA/NO	≥1 (I)	≥1 (I)	≥1 (I)	≥1 (I)
	DETA/NO	≥1 (I)	≥1 (I)	≥1 (I)	≥1 (I)
5-Fluorocytosine	MD3	≥1 (I)	≥1 (I)	≥1 (I)	≥1 (I)
	SPER/NO	≥1 (I)	≥1 (I)	≥1 (I)	≥1 (I)
	DPTA/NO	≥1 (I)	≥1 (I)	≥1 (I)	≥1 (I)
	DETA/NO	≥1 (I)	≥1 (I)	≥1 (I)	≥1 (I)
Amphotericin B	MD3	≥1 (I)	≥1 (I)	≥1 (I)	≥1 (I)
	SPER/NO	≥1 (I)	≥1 (I)	≥1 (I)	≥1 (I)
	DPTA/NO	≥1 (I)	≥1 (I)	≥1 (I)	≥1 (I)
	DETA/NO	≥1 (I)	≥1 (I)	≥1 (I)	≥1 (I)
Butenafine	MD3	≥1 (I)	≥1 (I)	0.500 (A)	≥1 (I)
	SPER/NO	≥1 (I)	≥1 (I)	0.562 (A)	≥1 (I)
	DPTA/NO	≥1 (I)	≥1 (I)	≥1 (I)	≥1 (I)
	DETA/NO	≥1 (I)	≥1 (I)	0.500 (A)	≥1 (I)
Miconazole	MD3	0.530 (A)	0.562 (A)	≥1 (I)	≥1 (I)
	SPER/NO	0.265 (S)	0.375 (S)	0.500 (S)	≥1 (I)
	DPTA/NO	≥1 (I)	0.625 (A)	0.375 (S)	≥1 (I)
	DETA/NO	0.375 (S)	≥1 (I)	0.562 (A)	≥1 (I)

^a^ Checkerboard assay results determined from n ≥ 3 experiments.

**Table 4:** Selectivity indexes of NO donors for each strain of *C. albicans*, *C. auris*, *C. neoformans*, and *A. fumigatus*.

Fungi	Strain	Selectivity Index			
		MD3	SPER/NO	DPTA/NO	DETA/NO
*C. albicans*	ATCC MYA-2876	267	164	35	383
	ATCC 18804	400	137	28	77
	ATCC 14053	107	82	28	77
*C. auris*	ATCC MYA-5000	533	164	28	77
	ATCC MYA-5001	800	82	35	1595
	ATCC MYA-5003	400	164	57	154
*C. neoformans*	ATCC 208821	533	41	18	191
	ATCC MYA-4566	842	1079	28	613
	ATCC MYA-4567	800	263	28	154
*A. fumigatus*	ATCC 1022	228	293	14	150

## Data Availability

Data are contained within the article and Supplementary Materials.

## Supplementary Materials

The following supporting information can be downloaded at: https://www.mdpi.com/article/10.3390/jof10050308/s1, Figure S1: Real-time NO release of (A) MD3, (B) SPER/NO, (C) DPTA/NO, and (D) DETA/NO; Table S1: Minimum inhibitory concentrations (MIC) of commercial antifungals against *C. albicans*, *C. auris*, *C. neoformans*, and *A. fumigatus*; Table S2: MIC of base scaffolds SPER, DPTA, and DETA; Figure S2: Scanning electron micrographs of (A) untreated *C. neoformans* and *C. neoformans* treated with (B) MD3, (C) SPER/NO, (D) DPTA/NO, and (E) DETA/NO at 10x the MIC for each compound; Figure S3: Scanning electron micrographs of (A) spermine treated *C. albicans* and (B) spermine treated *C. auris*; Table S3: IC_50_ values of NO-releasing small molecules against AIR-100 EpiAirway Tissues.

## References

[1] Hasim S., Coleman J.J. (2019). Targeting the Fungal Cell Wall: Current Therapies and Implications for Development of Alternative Antifungal Agents. Future Med. Chem..

[2] Fausto A., Rodrigues M.L., Coelho C. (2019). The Still Underestimated Problem of Fungal Diseases Worldwide. Front. Microbiol..

[3] Ksiezopolska E., Gabaldón T. (2018). Evolutionary Emergence of Drug Resistance in Candida Opportunistic Pathogens. Genes.

[4] Benedict K., Whitham H.K., Jackson B.R. (2022). Economic Burden of Fungal Diseases in the United States. Open Forum Infect. Dis..

[5] Kainz K., Bauer M.A., Madeo F., Carmona-Gutierrez D. (2020). Fungal Infections in Humans: The Silent Crisis. Microb. Cell.

[6] World Health Organization (2022). WHO Fungal Priority Pathogens List to Guide Research, Development and Public Health Action.

[7] Meis J.F., Chowdhary A., Rhodes J.L., Fisher M.C., Verweij P.E. (2016). Clinical Implications of Globally Emerging Azole Resistance in Aspergillus Fumigatus. Philos. Trans. R. Soc. B Biol. Sci..

[8] Jeanvoine A., Rocchi S., Bellanger A.P., Reboux G., Millon L. (2020). Azole-Resistant Aspergillus Fumigatus: A Global Phenomenon Originating in the Environment?. Med. Mal. Infect..

[9] *C. neoformans* Infection|Fungal Diseases|CDC. https://www.cdc.gov/fungal/diseases/cryptococcosis-neoformans/index.html.

[10] Perlin D.S., Rautemaa-Richardson R., Alastruey-Izquierdo A. (2017). The Global Problem of Antifungal Resistance: Prevalence, Mechanisms, and Management. Lancet Infect. Dis..

[11] WHO Fungal Priority Pathogens List to Guide Research, Development and Public Health Action. https://www.who.int/publications/i/item/9789240060241.

[12] *Candida auris*|*Candida auris*|Fungal Diseases| CDC. https://www.cdc.gov/fungal/candida-auris/index.html.

[13] Dixon D.M., Walsh T.J. (1996). Antifungal Agents. Med. Microbiol..

[14] Szymański M., Chmielewska S., Czyżewska U., Malinowska M., Tylicki A. (2022). Echinocandins—Structure, Mechanism of Action and Use in Antifungal Therapy. J. Enzym. Inhib. Med. Chem..

[15] Vermes A., Guchelaar H.J., Dankert J. (2000). Flucytosine: A Review of Its Pharmacology, Clinical Indications, Pharmacokinetics, Toxicity and Drug Interactions. J. Antimicrob. Chemother..

[16] Wall G., Lopez-Ribot J.L. (2020). Current Antimycotics, New Prospects, and Future Approaches to Antifungal Therapy. Antibiotics.

[17] Gray K.C., Palacios D.S., Dailey I., Endo M.M., Uno B.E., Wilcock B.C., Burke M.D. (2012). Amphotericin Primarily Kills Yeast by Simply Binding Ergosterol. Proc. Natl. Acad. Sci. USA.

[18] Chudzik B., Bonio K., Dabrowski W., Pietrzak D., Niewiadomy A., Olender A., Malodobry K., Gagoś M. (2019). Synergistic Antifungal Interactions of Amphotericin B with 4-(5-Methyl-1,3,4-Thiadiazole-2-Yl) Benzene-1,3-Diol. Sci. Rep..

[19] Boyce K.J., Andrianopoulos A. (2015). Fungal Dimorphism: The Switch from Hyphae to Yeast Is a Specialized Morphogenetic Adaptation Allowing Colonization of a Host. FEMS Microbiol. Rev..

[20] Overview of Fungal Infections—Infections—Merck Manuals Consumer Version. https://www.merckmanuals.com/home/infections/fungal-infections/overview-of-fungal-infections.

[21] Treatment and Management of Infections and Colonization|*Candida auris*|Fungal Diseases|CDC. https://www.cdc.gov/fungal/candida-auris/c-auris-treatment.html.

[22] Antifungal Susceptibility Testing and Interpretation *Candida auris*|Fungal Diseases|CDC. https://www.cdc.gov/fungal/candida-auris/c-auris-antifungal.html.

[23] Rauseo A.M., Coler-Reilly A., Larson L., Spec A. (2020). Hope on the Horizon: Novel Fungal Treatments in Development. Open Forum Infect. Dis..

[24] Roemer T., Krysan D.J. (2014). Antifungal Drug Development: Challenges, Unmet Clinical Needs, and New Approaches. Cold Spring Harb. Perspect. Med..

[25] Rouillard K.R., Novak O.P., Pistiolis A.M., Yang L., Ahonen M.J.R., McDonald R.A., Schoenfisch M.H. (2021). Exogenous Nitric Oxide Improves Antibiotic Susceptibility in Resistant Bacteria. ACS Infect. Dis..

[26] Maloney S.E., Mcgrath K.V., Ahonen M.J.R., Soliman D.S., Feura E.S., Hall H.R., Wallet S.M., Maile R., Schoenfisch M.H. (2020). Nitric Oxide-Releasing Hyaluronic Acid as an Antibacterial Agent for Wound Therapy. Biomacromolecules.

[27] Stasko N., McHale K., Hollenbach S.J., Martin M., Doxey R. (2018). Nitric Oxide-Releasing Macromolecule Exhibits Broad-Spectrum Antifungal Activity and Utility as a Topical Treatment for Superficial Fungal Infections. Antimicrob. Agents Chemother..

[28] Macherla C., Sanchez D.A., Ahmadi M.S., Vellozzi E.M., Friedman A.J., Nosanchuk J.D., Martinez L.R. (2012). Nitric Oxide Releasing Nanoparticles for Treatment of Candida Albicans Burn Infections. Front. Microbiol..

[29] Liu L., Pan X., Liu S., Hu Y., Ma D. (2021). Near-Infrared Light-Triggered Nitric Oxide Release Combined with Low-Temperature Photothermal Therapy for Synergetic Antibacterial and Antifungal. Smart Mater. Med..

[30] Madariaga-Venegas F., Fernández-Soto R., Duarte L.F., Suarez N., Delgadillo D., Jara J.A., Fernández-Ramires R., Urzia B., Molina-Berríos A. (2017). Characterization of a Novel Antibiofilm Effect of Nitric Oxide-Releasing Aspirin (NCX-4040) on Candida Albicans Isolates from Denture Stomatitis Patients. PLoS ONE.

[31] Vargas-Cruz N., Reitzel R.A., Rosenblatt J., Chaftari A.M., Dib R.W., Hachem R., Kontoyiannis D.P., Raad I.I. (2019). Nitroglycerin-Citrate-Ethanol Catheter Lock Solution Is Highly Effective for In Vitro Eradication of Candida Auris Biofilm. Antimicrob. Agents Chemother..

[32] Hetrick E.M., Shin J.H., Stasko N.A., Johnson C.B., Wespe D.A., Holmuhamedov E., Schoenfisch M.H. (2008). Bactericidal Efficacy of Nitric Oxide-Releasing Silica Nanoparticles. ACS Nano.

[33] Carpenter A.W., Worley B.V., Slomberg D.L., Schoenfisch M.H. (2012). Dual Action Antimicrobials: Nitric Oxide Release from Quaternary Ammonium-Functionalized Silica Nanoparticles. Biomacromolecules.

[34] Carpenter A.W., Reighard K.P., Saavedra J.E., Schoenfisch M.H. (2013). O2-Protected Diazeniumdiolate-Modified Silica Nanoparticles for Extended Nitric Oxide Release from Dental Composites. Biomater. Sci..

[35] McElhaney-Feser G.E., Raulli R.E., Cihlar R.L. (1998). Synergy of Nitric Oxide and Azoles against Candida Species In Vitro. Antimicrob. Agents Chemother..

[36] (2015). Reference Method for Broth Dilution Antifungal Susceptibility Testing of Yeasts.

[37] European Committee on Antimicrobial Susceptibility Testing Breakpoint Tables for Interpretation of MICs for Antifungal Agents. https://www.eucast.org/astoffungi/clinicalbreakpointsforantifungals.

[38] Bellio P., Fagnani L., Nazzicone L., Celenza G. (2021). New and Simplified Method for Drug Combination Studies by Checkerboard Assay. MethodsX.

[39] Salama A.H. (2023). Study the Activity of Conjugated Antimicrobial Peptide WW-185 Against Clinically Important Bacteria. Pharmacia.

[40] Yang L., Schoenfisch M.H. (2018). Nitric Oxide-Releasing Hyperbranched Polyaminoglycosides for Antibacterial Therapy. ACS Appl. Bio Mater..

[41] Storm W.L., Schoenfisch M.H. (2013). Nitric Oxide-Releasing Xerogels Synthesized from N-Diazeniumdiolate-Modified Silane Precursors. ACS Appl. Mater. Interfaces.

[42] Reighard K.P., Schoenfisch M.H. (2015). Antibacterial Action of Nitric Oxide-Releasing Chitosan Oligosaccharides against Pseudomonas Aeruginosa under Aerobic and Anaerobic Conditions. Antimicrob. Agents Chemother..

[43] Rouillard K.R., Markovetz M.R., Bacudio L.G., Hill D.B., Schoenfisch M.H. (2020). Pseudomonas Aeruginosa Biofilm Eradication via Nitric Oxide-Releasing Cyclodextrins. ACS Infect. Dis..

[44] Hrabie J.A., Keefer L.K. (2002). Chemistry of the Nitric Oxide-Releasing Diazeniumdiolate (“nitrosohydroxylamine”) Functional Group and Its Oxygen-Substituted Derivatives. Chem. Rev..

[45] (2021). Folk, D; Anderson, R.G.; Simons, J.K.; Ahonen, M.J.R.; McDonald, R.A. Nitric Oxide-Releasing Antibacterial Compounds, Formulations, and Methods Pertaining Thereto. International Patent.

[46] Różalska B., Sadowska B., Budzyńska A., Bernat P., Różalska S. (2018). Biogenic Nanosilver Synthesized in Metarhizium Robertsii Waste Mycelium Extract—As a Modulator of Candida Albicans Morphogenesis, Membrane Lipidome and Biofilm. PLoS ONE.

[47] Yang Y., Wang C., Zhuge Y., Zhang J., Xu K., Zhang Q., Zhang H., Chen H., Chu M., Jia C. (2019). Photodynamic Antifungal Activity of Hypocrellin a against Candida Albicans. Front. Microbiol..

[48] Zhou X., He P. (2011). Improved Measurements of Intracellular Nitric Oxide in Intact Microvessels Using 4,5-Diaminofluorescein Diacetate. Am. J. Physiol. Heart Circ. Physiol..

[49] Neilson L., Mankus C., Thorne D., Jackson G., DeBay J., Meredith C. (2015). Development of an in Vitro Cytotoxicity Model for Aerosol Exposure Using 3D Reconstructed Human Airway Tissue; Application for Assessment of e-Cigarette Aerosol. Toxicol. In Vitro.

[50] Li Z., Lu G., Meng G. (2019). Pathogenic Fungal Infection in the Lung. Front. Immunol..

[51] Mota Fernandes C., Dasilva D., Haranahalli K., McCarthy J.B., Mallamo J., Ojima I., Del Poeta M. (2021). The Future of Antifungal Drug Therapy: Novel Compounds and Targets. Antimicrob. Agents Chemother..

[52] Cánovas D., Marcos J.F., Marcos A.T., Strauss J. (2016). Nitric Oxide in Fungi: Is There NO Light at the End of the Tunnel?. Current Genetics.

[53] Privett B.J., Nutz S.T., Schoenfisch M.H. (2010). Efficacy of Surface-Generated Nitric Oxide against Candida Albicans Adhesion and Biofilm Formation. Biofouling.

[54] Weinberger B., Laskin D.L., Heck D.E., Laskin J.D. (2001). The Toxicology of Inhaled Nitric Oxide. Toxicol. Sci..

[55] Ahmadi M.S., Lee H.H., Sanchez D.A., Friedman A.J., Tar M.T., Davies K.P., Nosanchuk J.D., Martinez L.R. (2016). Sustained Nitric Oxide-Releasing Nanoparticles Induce Cell Death in Candida Albicans Yeast and Hyphal Cells, Preventing Biofilm Formation In Vitro and in a Rodent Central Venous Catheter Model. Antimicrob. Agents Chemother..

[56] Hrabie J.A., Klose J.R., Wink D.A., Keefer L.K. (1993). New Nitric Oxide-Releasing Zwitterions Derived from Polyamines. J. Org. Chem..

[57] Jeong H., Park S., Park K., Kim M., Hong J. (2020). Sustained Nitric Oxide-Providing Small Molecule and Precise Release Behavior Study for Glaucoma Treatment. Mol. Pharm..

[58] Fink S., Burmester A., Hipler U.C., Neumeister C., Götz M.R., Wiegand C. (2022). Efficacy of Antifungal Agents against Fungal Spores: An In Vitro Study Using Microplate Laser Nephelometry and an Artificially Infected 3D Skin Model. Microbiologyopen.

[59] Seidl H.P., Jäckel A., Müller J., Schaller M., Borelli C., Polak A. (2015). Sporicidal Effect of Amorolfine and Other Antimycotics Used in the Therapy of Fungal Nail Infections. Mycoses.

[60] Tillmann A., Gow N.A.R., Brown A.J.P. (2011). Nitric Oxide and Nitrosative Stress Tolerance in Yeast. Biochem. Soc. Trans..

[61] Farrugia G., Balzan R. (2012). Oxidative Stress and Programmed Cell Death in Yeast. Front. Oncol..

[62] Ullmann B.D., Myers H., Chiranand W., Lazzell A.L., Zhao Q., Vega L.A., Lopez-Ribot J.L., Gardner P.R., Gustin M.C. (2004). Inducible Defense Mechanism against Nitric Oxide in Candida Albicans. Eukaryot. Cell.

[63] Chen H., Zhou X., Ren B., Cheng L. (2020). The Regulation of Hyphae Growth in Candidaï¿¿albicans. Virulence.

[64] Koch B., Barugahare A.A., Lo T.L., Huang C., Schittenhelm R.B., Powell D.R., Beilharz T.H., Traven A. (2018). A Metabolic Checkpoint for the Yeast-to-Hyphae Developmental Switch Regulated by Endogenous Nitric Oxide Signaling. Cell Rep..

[65] Van Dijck P., Sjollema J., Cammue B.P.A., Lagrou K., Berman J., d’Enfert C., Andes D.R., Arendrup M.C., Brakhage A.A., Calderone R. (2018). Methodologies for In Vitro and In Vivo Evaluation of Efficacy of Antifungal and Antibiofilm Agents and Surface Coatings against Fungal Biofilms. Microb. Cell.

[66] Sadozai S.K., Khan S.A., Baseer A., Ullah R., Zeb A., Schneider M. (2022). In Vitro, Ex Vivo, and In Vivo Evaluation of Nanoparticle-Based Topical Formulation against Candida Albicans Infection. Front. Pharmacol..

