# Supplementary material for: Impact of Nitric Oxide-Release Kinetics on Antifungal Activity
Source: J Fungi (Basel). 2024 Apr 24;10(5):308. doi: 10.3390/jof10050308 (PMC11121837; doi:10.3390/jof10050308)
Supplement: Supplementary file 1 [file jof-10-00308-s001.zip › jof-2941720-supplementary.pdf]

Impact of Nitric Oxide-Release Kinetics on Antifungal Activity

Supplementary Information

Quincy E. Grayton,[1] Ivie L. Conlon,[1] Christopher A. Broberg,[1] and Mark H. Schoenfisch[1,2*]

[1]Department of Chemistry and [2]Eshelman School of Pharmacy, University of North Carolina at Chapel Hill, North Carolina, USA

[*]To whom correspondence should be addressed: schoenfisch@unc.edu

Contents

Figure S1. Real-time NO release of (A) MD3, (B) SPER/NO, (C) DPTA/NO, and (D) DETA/NO. 2

Table S1. Minimum inhibitory concentrations (MIC) of commercial antifungals against *C. albicans*, *C. auris*, *C. neoformans*, and *A. fumigatus*. 3

Table S2. MIC of base scaffolds SPER, DPTA, and DETA. 4

Figure S3. Scanning electron micrographs of (A) spermine treated *C. albicans* and (B) spermine treated *C. auris*. 5

Figure S2. Scanning electron micrographs of (A) untreated *C. neoformans* and *C. neoformans* treated with (B) MD3, (C) SPER/NO, (D) DPTA/NO, and (E) DETA/NO at 10x the MIC for each compound. 5

Table S3. IC50 values of NO-releasing small molecules against AIR-100 EpiAirway Tissues. 6

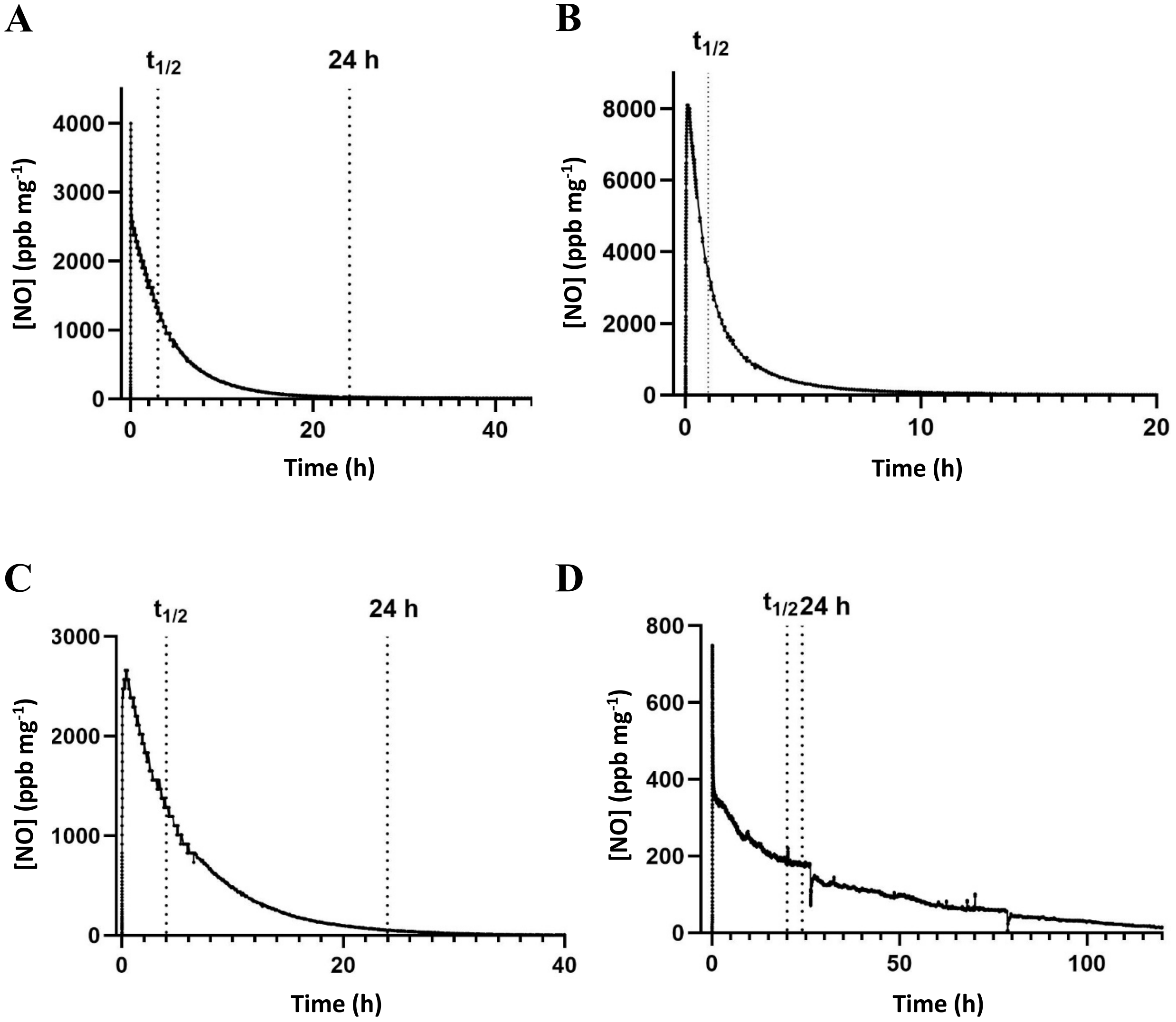

Figure S1. Real-time NO release of (A) MD3, (B) SPER/NO, (C) DPTA/NO, and (D) DETA/NO. Half-life of NO release is indicated by $t_{1/2}$.

Table S1. Minimum inhibitory concentrations (MIC) of commercial antifungals against *C. albicans*, *C. auris*, *C. neoformans*, and *A. fumigatus*.[a]

Strains	MIC ($\mu g\ mL^{-1}$)					
	**CAS**	**FLC**	**5FC**	**AMB**	**BUT**	**MICO**
C. albicans (ATCC MYA-2876)	0.25	>32*	0.125	0.25	>4000	60
C. auris (ATCC MYA-5001)	>32*	>32*	2	0.5	>4000	4
C. neoformans (ATCC 208821)	32	4	4	0.195	0.3	0.5
A. fumigatus (ATCC 1022)	125	>125	>62.5	2.5*	>500	>500

[a]MIC determined from n ≥ 3 experiments. CAS = caspofungin, FLC = fluconazole, 5FC = 5-fluorocytosine, AMB = amphotericin B, BUT = butenafine, MICO = miconazole. *Denotes resistance to antifungal.

Table S2. MIC of base scaffolds SPER, DPTA, and DETA.[a]

Strains	MIC (mg mL^{-1})		
	SPER	DPTA	DETA
Candida albicans			
ATCC MYA-2876	>20	20	>20
ATCC 18804	>20	20	>20
ATCC 14053	20	20	>20
Candida auris			
ATCC MYA-5000	>20	>20	>20
ATCC MYA-5001	>20	>20	>20
ATCC MYA-5003	>20	>20	>20
Cryptococcus neoformans			
ATCC 208821	5	5	>20
ATCC MYA-4566	2.5	5	>20
ATCC MYA-4567	2.5	2.5	10

[a]Determined from $n \geq 3$ experiments.

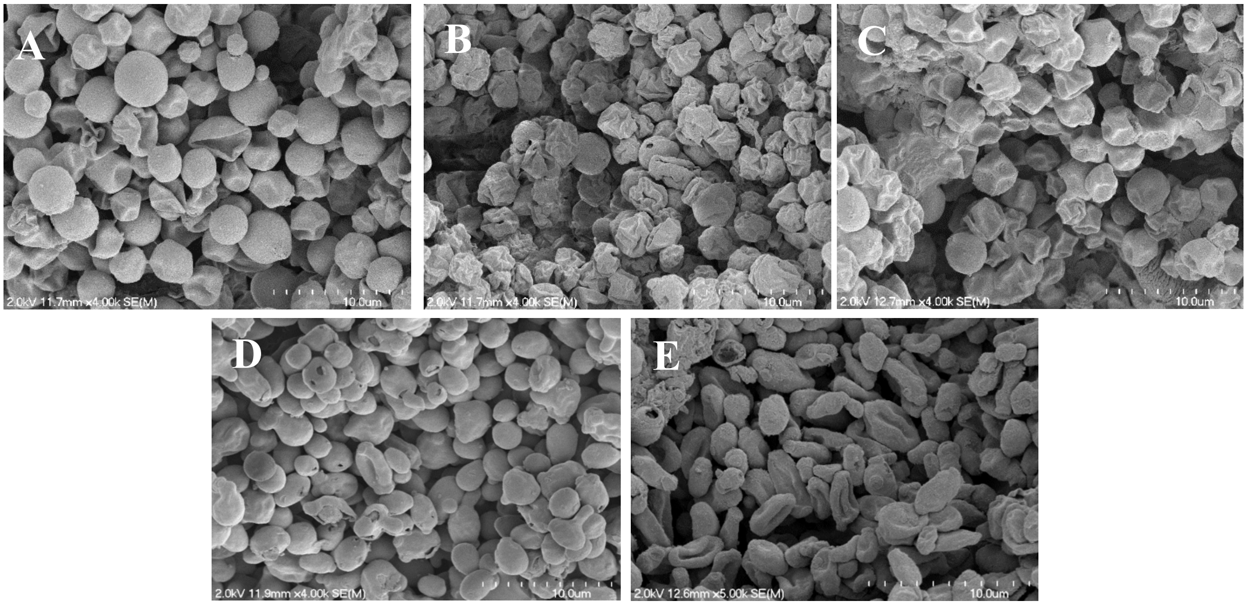

Figure S2. Scanning electron micrographs of (A) untreated *C. neoformans* and *C. neoformans* treated with (B) MD3, (C) SPER/NO, (D) DPTA/NO, and (E) DETA/NO at 10x the MIC for each compound. Images are representative of n ≥ 3 separate experiments.

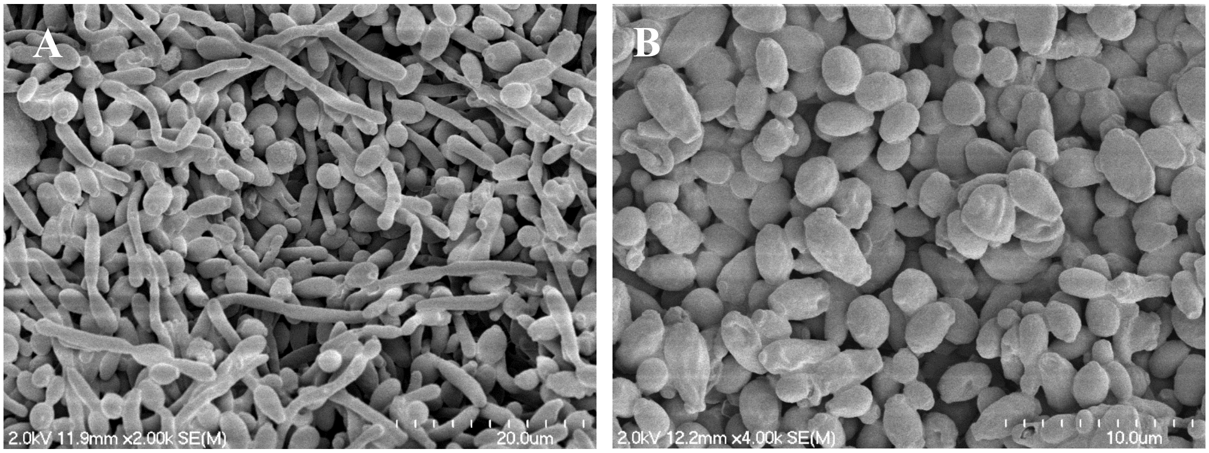

Figure S3. Scanning electron micrographs of (A) spermine treated *C. albicans* and (B) spermine treated *C. auris*. Treatments concentrations were 10x the MIC for each fungal strain. Images are representative of n ≥ 3 experiments.

Table S3. IC50 values of NO-releasing small molecules against AIR-100 EpiAirway Tissues.[a]

**NO donor**	**IC_{50} (µg mL^{-1})**
MD3	16,000 ± 1,000
SPER/NO	20,000 ± 4,000
DPTA/NO	18,000 ± 2,000
DETA/NO	48,000 ± 7,000

[a]IC_{50} determined from n ≥ 3 experiments.
